# Preliminary effectiveness and feasibility of ASHA-led mobile health intervention for diabetes care in Indian primary health care settings

**DOI:** 10.1038/s41598-025-20728-w

**Published:** 2025-10-21

**Authors:** Abhinav Bassi, Varun Arora, Sumaiya Arfin, Oommen John, Kavita Yadav, Devarsetty Praveen, O. P. Kalra, S. V. Madhu, Vivekanand Jha

**Affiliations:** 1 The George Institute for Global Health, UNSW, New Delhi, India; 2 Pandit Bhagwat Dayal Sharma, University of Health Sciences, Rohtak, Haryana India; 3 Amsterdam UMC Doctoral School, Amsterdam, Netherlands; 4 Manipal Academy of Higher Education, Manipal, Karnataka India; 5 University of New South Wales, Sydney, NSW Australia; 6 Santosh Medical College & Hospital, Ghaziabad, Uttar Pradesh India; 7 University College of Medical Sciences, Delhi, India; 8 Imperial College London, London, UK; 9 School of Public Health, Imperial College, London, UK

**Keywords:** Diabetes, MHealth, ASHA, Blood glucose, Frontline health workers, Clinical decision support system, Endocrinology, Health care, Medical research, Diabetes

## Abstract

Diabetes management in resource-limited settings faces challenges in screening, guideline-based treatment, and healthcare access. The IMPACT Diabetes study evaluated a community-based, technology-enabled task-shifting intervention for diabetes care in India. A cluster randomized controlled trial was conducted in 16 villages/peri-urban areas across 8 primary health centers (PHCs) in two states in India. Accredited Social Health Activists (ASHAs) screened 1,785 community participants, identifying 418 individuals with diabetes. The intervention group received nine months of CDSS-supported care delivered by ASHAs under physician supervision, while the control group received usual care. The primary outcome was the proportion of participants achieving ≥ 0·5% reduction in glycated haemoglobin (HbA1c) from baseline. Secondary outcomes included healthcare utilization and medication adherence. A significantly higher proportion of intervention participants achieved HbA1c reduction ≥ 0·5% compared to the control group (21.8% vs. 10.3%, *p* < 0.05). Intervention participants had more frequent physician visits (85·0% vs. 29·8%), higher glucose-lowering medication adherence (63·0% vs. 43·1%, *p* < 0·05), and better engagement with diabetes management practices. Qualitative findings demonstrated that the intervention was acceptable and feasible for patients, ASHAs, and physicians, empowering ASHAs in chronic disease care. This study demonstrates that task-shifting and digital health tools can improve diabetes outcomes in low-resource settings. Future research should explore long-term sustainability and cost-effectiveness.

## Introduction

India faces a rapidly growing diabetes epidemic, with a significant impact on individuals, families, and the healthcare system^1,2^. In 2021, India had the second-highest number of adults living with diabetes globally, with an estimated 74.2 million adults aged 20 to 79 years affected^3^. This number is projected to exceed 124 million by 2045^3^. India accounts for the third-highest number of deaths attributed to diabetes globally, with an estimated 0.33 million deaths annually. These deaths predominantly arise from diabetes-related complications, such as cardiovascular disease (CVD), kidney disease, and peripheral vascular disease^3^. The reported number of death attribution to diabetes may also be influenced by coding practices that do not capture all causes of death.

Several challenges hinder diabetes management in countries with weak healthcare systems. These include a shortage of trained healthcare professionals and limited access to screening programs, essential medicines, and diagnostics^4–6^. This is exacerbated for those living in rural areas and without access to health insurance^7^. While India has implemented diabetes screening programs, their effective implementation remains a challenge, leading to delayed diagnosis and a high risk of complications^8^. Even after diagnosis, access to consistent, guideline-based care remains limited^9^.

Innovative approaches are, therefore, needed. The Indian Government’s initiative to establish Health and Wellness Centers (HWCs) as hubs for comprehensive primary health care offers a promising platform in this regard^10^. However, effective implementation requires strategies for optimizing existing resources and leveraging technology to improve access and quality of care. Task-shifting, where actions traditionally performed by physicians are delegated to trained nurses or community health workers like Accredited Social Health Activists (ASHA), has shown promise in improving health outcomes^11–13^.

There already exists literature evaluating the use of CHWs for diabetes management, especially in low-income and middle-income countries (LMICs) facing physician shortages^14^. While these interventions show moderate effectiveness in diabetes management in LMIC settings, systematic reviews indicate that interventions led specifically by community health workers do not show meaningful reductions in HbA1c unless adequately supported by additional resources^15^. Digital technology, particularly mobile health (mHealth) tools, are important in this regard. Clinical decision support systems (CDSS) provide real-time guidance on diagnosis, treatment, and monitoring, enhancing healthcare workers’ knowledge and skills, and promoting adherence to clinical guidelines^16–19^. In a study in rural Indonesia, use of this approach resulted in an increase in the use of appropriate preventive CVD medications and improved blood pressure control in individuals with hypertension^20^. However, in India, where persistent system-level challenges such as low digital literacy, infrastructure gaps, under-resourcing of health workers, and urban–rural and gender disparities are prevalent, it is critical that such systems be rigorously tested and adapted to ensure their effectiveness and scalability in the local context.

The IMPACT Diabetes study builds upon these concepts by developing and evaluating a multifaceted mobile technology–supported primary healthcare intervention that combines task-shifting with a mobile-based CDSS to improve diabetes identification and management within the existing primary healthcare system in India.

## Methods

### Study objectives

The IMPACT Diabetes study was a cluster randomized controlled trial designed to evaluate the preliminary effectiveness of this intervention in the management of diabetes in the Indian primary healthcare setting, as assessed by the difference in glycated hemoglobin (HbA1c) between baseline and end line assessments in the two groups. The study also aimed to determine the acceptability and feasibility of the intervention from the perspectives of patients, ASHAs, and physicians through qualitative interviews and focus group discussions (FGD). The study design has already been published^21^.

### Study setting

The study was conducted in 16 villages/peri-urban areas in eight PHCs of two districts: Guntur, Andhra Pradesh, and Rohtak, Haryana. The selection ensured the representation of diverse geographies, socio-economic conditions, healthcare access levels, and ASHA workload patterns. The detailed process of selecting PHCs and ASHAs is described in the design paper^21^.

### Participant eligibility and recruitment

Within each village/area, one ASHA was randomly selected to participate in the study. ASHA were tasked with screening approximately 100 community members aged 30 years or older within their assigned areas. Participants were eligible for the study if they resided within the selected area, were 30 years of age or older, and provided written informed consent. Subjects with any physical or intellectual disability that prevented follow-up or participation in the study procedures, pregnancy, and an intention to relocate from the community during the study period were excluded from the study.

### Study procedures

#### Intervention group

The IMPACT Diabetes intervention was a multi-component strategy that included a mobile tablet application-based CDSS called *SMARThealth* (Systematic Medical Appraisal, Referral and Treatment)^22^. This application provided ASHAs and PHC physicians with a comprehensive platform for diabetes screening, diagnosis, management, and follow-up, incorporating evidence-based guidelines and algorithms to assist with clinical decision-making^23–25^. To equip ASHAs for their expanded responsibilities, a comprehensive training program consisting of in-person training, supervised field practice, and subsequent booster sessions were provided. Details of training are already published^21^. Measures were taken to prevent overburdening ASHAs and maintain their motivation, including provision of user-friendly tablets and diagnostic tools to streamline data collection and CDSS access, regular discussion on workload, and ongoing support from the study team. PHC physicians received guidance on utilizing the electronic data and CDSS for informed management decisions and interpreting the data collected by ASHAs, enabling them to provide patient-specific recommendations for medication management and lifestyle modifications. This approach promoted task-sharing between ASHAs and physicians, with ASHAs taking on a greater role in diabetes identification and basic management under the supervision of physicians.

#### Control group

Participants in the control group received usual care according to the existing public health system, provided by PHC doctors or private physicians. During screening visits, ASHAs provided general information about diabetes and its complications. Those who screened positive were referred for confirmatory testing and advised to visit local PHCs or the private physician of their choice.

## Data collection and measures

### Preliminary effectiveness

Data were collected by ASHAs from all participants, including demographic information (age, gender, marital status, educational qualification), anthropometric measurements [height, weight, and body mass index (BMI)], BP [measured using Bluetooth-enabled digital BP monitors (A&D UA-767 Plus)], and capillary blood glucose (measured using Abbott FreeStyle Optium Neo monitors). Information was also collected on medical history (self-reported history of diabetes, hypertension, CVD, and other relevant conditions), family history of diabetes and CVD, behavioral risk factors (such as physical activity and tobacco use), and current medication use, including any anti-hypertensive, anti-platelet, lipid-lowering, and blood glucose-lowering medications.

ASHAs conducted monthly follow-up visits for nine months for all participants, collecting data on blood glucose levels, BP, and treatment adherence, assessed through participant self-report to minimize recall bias. This information was uploaded to the cloud and was accessed by physicians to plan the management. After reviewing data and lab reports, physicians entered information about the medications prescribed to each participant. HbA1c testing was conducted by accredited labs at baseline and end line. Labs were blinded to the treatment allocation.

To ensure data quality, automated checks were implemented within the application to validate the data and flag values outside acceptable ranges. A monitoring team continuously reviewed the data collected from the field, providing regular feedback to ASHAs and physicians, and identified areas where retraining or booster training sessions were needed.

### Acceptability and feasibility

Qualitative evaluation was conducted at the end of the study to assess the acceptability and implementation feasibility of the intervention through FGDs and semi-structured interviews with stakeholders. Participants were purposively selected to ensure representation and to capture a range of perspectives and experiences, including individuals with varying ages, genders, and duration of diabetes (for participants).

Semi-structured interviews were conducted with all physicians, four patients, and five ASHAs. Four FGDs were conducted: two with ASHAs (four participants each) and two with persons with diabetes (four males and four females each).

Semi-structured interview guides and FGD guides were developed based on a review of relevant literature and the research questions outlined. Participant experiences with the intervention, staff roles and responsibilities; patient, ASHA, and PHC physician satisfaction with the tablet-based CDSS; the impact of the CDSS on staff knowledge and skills; and the impact of the CDSS on usual work routines were explored. Interviews and FGDs were conducted by trained researchers.

## Analysis

### Preliminary effectiveness

Mean and standard deviation (SD) were calculated to summarize continuous variables. Chi-square tests were used to assess the association between categorical variables. T-tests were used to compare the means of continuous variables. Detailed sample size calculations have been published in our design paper^21^. The primary outcome was the between-group difference in the proportion of participants with diabetes achieving a 0·5% reduction in HbA1c. Secondary outcomes included the proportion of individuals visiting a physician and adherence to blood glucose-lowering medications. For categorical secondary outcomes, including PHC physician visits, private doctor visits, and the proportion of participants on glucose-lowering medicines, we used Fisher’s exact test. A p-value of less than 0·05 was considered statistically significant.

For the qualitative component, data collection continued until thematic saturation was achieved, ensuring an understanding of acceptability and feasibility.

### Acceptability and feasibility

Audio recordings were transcribed verbatim and translated into English by bilingual researchers fluent in Telugu/Hindi and English. Thematic analysis was employed to identify, analyze, and report patterns, drawing on the methods outlined by Patton^26^. This involved familiarization with the data through repeated reading of the transcripts, systematic coding to identify meaningful segments related to the research questions and emergent themes, grouping codes into broader themes and sub-themes, and reviewing and refining themes to ensure they accurately reflected the data. Data were analyzed using NVivo 13.

### Ethical considerations

Ethical approvals were obtained from the Institutional Ethics Committees of the Centre for Chronic Disease Control, New Delhi (FWA00012746), and PGIMS, Rohtak, Haryana (IEC/18/524). All study participants provided written informed consent. All methods were performed in accordance with the relevant guidelines and regulations, including the Declaration of Helsinki.

## Results

### Retention and follow-up

**Fig. 1:**
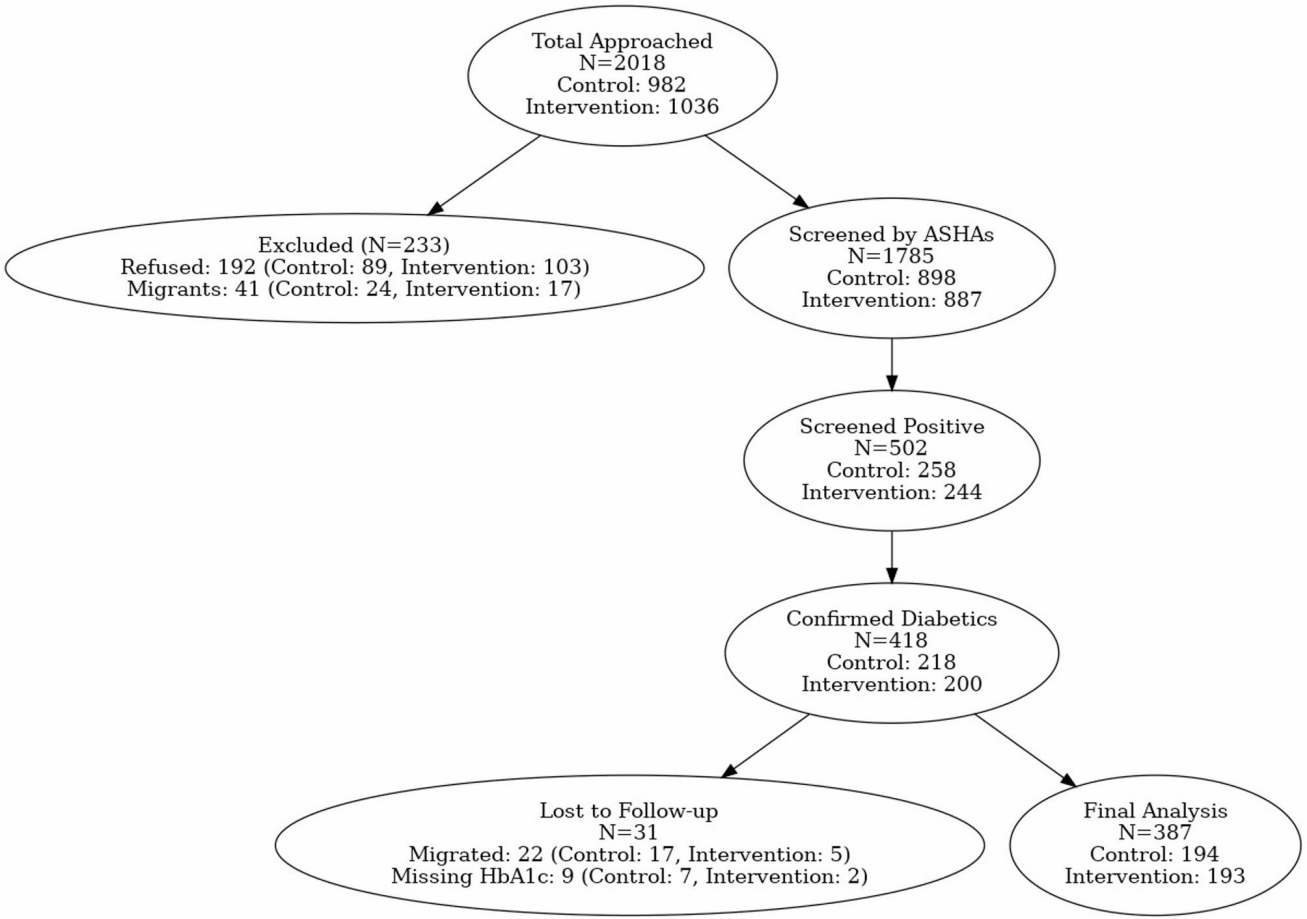
Study Flow. ASHA: Accredited Social Health Activist.

Recruitment was carried out between January and August 2019 in Guntur and between August 2019 and January 2020 in Rohtak. The end-of-study HbA1c measurement was completed at both sites by September 2020. Figure 1 outlines the study flow. A total of 2,018 community members were approached. After excluding those who refused or were likely to move, 1,785 were screened for diabetes. Of those, 502 screened positive and 418 were confirmed to have diabetes. A total of 387 (92·6%) participants (194 in the control group and 193 in the intervention group) completed the end-of-study HbA1c measurement. Of the 31 (7·4%) who could not complete the final HbA1c measurement, 22 had migrated out before the final assessment, and 9 had missing HbA1c values for other reasons.

### Baseline characteristics

Table 1 outlines the baseline characteristics of all participants. Overall, the groups were similar in baseline characteristics, except for current smoker proportions and history of angina/heart attack.

**Table 1:** Baseline characteristics of people with confirmed diabetics in the control and intervention arms.

	Intervention (*n* = 200)	Control (*n* = 218)	Total (*n* = 418)
Female	112 (56·0%)	120 (55·0%)	232 (55·5%)
Age (years), mean (SD)	56·9 (32·5)	55·8 (11·7)	56·3 (24·0)
Weight (kg), mean (SD)	65·9 (15·3)	64·8 (13·1)	65·3 (14·2)
BMI (kg/m²), mean (SD)	27·2 (18·9)	26·1 (4·7)	26·6 (13·5)
Underweight (< 18·5)	8 (4·0%)	13 (6·0%)	21 (5·0%)
Normal (18·5–22·9)	46 (23·0%)	36 (16·5%)	82 (19·6%)
Overweight (23·0–24·9)	35 (17·5%)	36 (16·5%)	71 (17·0%)
Obese (> 25)	111 (55·5%)	133 (61·0%)	244 (58·4%)
Tobacco Use			
Current smoker	23 (11·5%)	46 (21·1%)	69 (16·5%)
Past smoker ^a^	2 (1·0%)	3 (1·4%)	5 (1·2%)
Chewing (current)	5 (2·5%)	6 (2·8%)	11 (2·6%)
Past chewing ^b^	1 (0·5%)	0 (0·0%)	1 (0·2%)
Medical History			
Diabetes	105 (52·5%)	127 (58·3%)	232 (55·5%)
Angina/heart attack	10 (5·0%)	25 (11·5%)	35 (8·4%)
Stroke	4 (2·0%)	7 (3·2%)	11 (2·6%)
Hypertension	58 (29·0%)	71 (32·6%)	129 (30·9%)
Peripheral vascular disease	0 (0·0%)	2 (0·9%)	2 (0·5%)
Family History ^c^			
Diabetes	72 (36·0%)	60 (27·5%)	132 (31·6%)
Angina/heart attack	26 (13·0%)	30 (13·8%)	56 (13·4%)
Stroke	20 (10·0%)	24 (11·0%)	44 (10·5%)
Medication History ^d^			
Anti-hypertensive	56 (28·0%)	67 (30·7%)	123 (29·4%)
Anti-platelet	4 (2·0%)	12 (5·5%)	16 (3·8%)
Lipid-lowering	3 (1·5%)	3 (1·4%)	6 (1·4%)
Blood glucose-lowering	85 (42·5%)	103 (47·2%)	188 (45·0%)
Currently Hypertensive	106 (53·0%)	122 (56·0%)	228 (54·5%)
Blood Pressure (mmHg)			
Systolic (SBP), mean (SD)	130·7 (23·9)	127·7 (20·9)	129·2 (22·4)
Diastolic (DBP), mean (SD)	82·3 (12·5)	82·1 (11·0)	82·2 (11·8)
HbA1c (%), mean (SD)	8·5 (1·8)	8·4 (1·8)	8·4 (1·8)

^a^ Has not smoked tobacco in the past 12 months; ^b^ Has not chewed tobacco in the past 12 months; ^c^ First-degree relatives (father, mother, and siblings) diagnosed with the disease; ^d^ Currently on antihypertensive drugs or SBP ≥ 140 mmHg and/or DBP ≥ 90 mmHg;.

### Primary and secondary effectiveness outcomes

A higher proportion of patients in the intervention group achieved a clinically meaningful reduction in HbA1c (≥ 0·5%) compared to the control group (21·8% vs. 10·3%, *p* < 0·05, Table 2). The analysis of secondary outcomes (Table 2) reveals significant differences in healthcare utilization and medication use between the two groups. A greater proportion of the intervention group visited PHC physicians compared to the control group (85% vs. 29·8%, *p* < 0·001). The proportion of participants visiting private doctors was numerically higher in the control group (20·2% vs. 13·5%, *p* = 0·09). The proportion of participants on glucose-lowering medicines increased by 20·5% in the intervention group, whereas the control group showed a decrease of 4·1% (*p* < 0·001). Mean HbA1c decreased by 0·21% (SD = 0·72%) in the intervention group compared to 0·31% (SD = 0.67%) in the control group (*p* = 0·15). There was no difference in the HbA1c levels at the final visit between the intervention (8·6 ± 1·7) and the control (8·8 ± 1·9) groups.

**Table 2:** Primary and secondary outcomes: Intervention vs. Control Groups.

Outcomes	Intervention (*n* = 200)	Control (*n* = 218)	*P*-value
Proportion achieving ≥ 0·5% reduction in HbA1c	42 (21·8%)	20 (10·3%)	*p < 0·016*
Mean ASHA visits (SD)	3·5 (1·5)	0·0 (0·0)	
PHC physician visits	170 (85·0%)	65 (29·8%)	*p < 0·001*
Private doctor visits	27 (13·5%)	44 (20·2%)	0·09
On glucose lowering medicines at endline	126 (63·0%)	94 (43·1%)	*p < 0·001*
Change in glucose lowering medicines between baseline and endline (%)	+ 20·5%	−4·1%	*p < 0·001*
Mean HbA1c at endline (SD) ^#^	8·6 (1·7)	8·8 (1·9)	0·40

^#^ HbA1c Values at endline not available for 31 participants, final analysed sample of 387 (control *n* = 194, intervention *n* = 193).

## Acceptability

### Patient satisfaction with treatment and management recommendations

Participants appreciated the comprehensive and accessible care and highlighted the perceived high quality of care, comparable to private facilities, but without the cost.

*“We are getting treatment better*,* when the ASHA came to my house*,* and with the mobile tablet*,* I felt that I was getting treatment better than at a private hospital…even though I do not have the money to go to a private hospital*,* I am being taken care of…I also shared this with my brother who lives in Oman” (Participant*,* Interview*,* Female*,* 54*,* Rohtak*,* Haryana)*.

The integration of technology, such as ASHAs utilizing mobile tablets and blood glucose monitors during home visits, was received well by the participants.

*“It was very helpful that all my blood tests were being done at home. ASHAs who were only taking care of small babies and pregnant ladies were also helping us maintain our health.” (Participant*,* FGD*,* Male*,* 55*,* Guntur*,* Andhra Pradesh)*.

Participants valued the continuity of care facilitated by the CDSS, which ensured physicians were well-informed about their health status before consultations. One participant shared:

*“When I was sent to the doctor*,* the doctor already knew about me and had my reports. This was a great experience.” (Participant*,* FGD*,* Female*,* 60*,* Rohtak*,* Haryana)*.

Provision of long-term medications and comprehensive lifestyle advice also contributed to patient satisfaction and empowerment.

### ASHA and physician experiences with the intervention

ASHAs and physicians shared positive experiences and perceived the intervention as beneficial. ASHAs expressed a sense of empowerment and enhanced professional development. While initially apprehensive about using the mobile tablets and new diagnostic tools, they quickly gained confidence with the support of the training provided.

*“We were already doing a lot of work in communicable diseases and maternal and child health…but we did gain some visibility and recognition from carrying this mobile tablet…We learned to not only use this mobile tablet but also the BP instrument and the blood glucose testing instrument.” (ASHA*,* Interview*,* Female*,* Rohtak*,* Haryana)*.

The intervention equipped ASHAs with new skills and knowledge, expanding their capacity to address a wider range of health concerns and enhancing their professional standing.

*“When I first told my friend who is a lab technician at one of the PHCs that I would be doing blood glucose testing*,* they were surprised*,* and it made me feel proud that I am able to do more than height and weight measurement in the community…” (ASHA*,* Interview*,* Female*,* Guntur*,* Andhra Pradesh)*.

Physicians valued the system’s ability to provide readily accessible patient information, treatment recommendations, and lifestyle modification suggestions through the CDSS.

*“The mobile app is very well developed…It’s good to have a reminder of the options of the drug that could be given to the participants based on their condition…just by a scan of a code I get to see the patient history*,* and also I am suggested options for the treatment and lifestyle modification options as well.” (Physician*,* Interview*,* Female*,* Guntur*,* Andhra Pradesh)*.

### Satisfaction with training and capacity building

An important component of the IMPACT Diabetes intervention was the training and capacity building of the ASHAs and physicians.

*“The initial training was quite a new concept for us because we were not managing non-communicable diseases or diabetes in the past… It was good to hear and get trained from the study team. Now many of our misconceptions that the villagers or our community members have were clarified.” (ASHA*,* FGD*,* Female*,* Guntur*,* Andhra Pradesh)*.

The hands-on training component, which included demonstrations of blood glucose and BP measurement using the CDSS, was particularly valued. Demonstration of these skills to dignitaries and district officials fostered a sense of empowerment and recognition for their expanded role in healthcare delivery.

*“Traveling to Delhi to showcase my training to dignitaries from Australia and the UK was a truly rewarding experience.” (ASHA*,* Interview*,* Female*,* Rohtak*,* Haryana)*.

They recognized the value of their contribution to diabetes care and felt motivated to continue their efforts.

*“15–18 of the diabetics in my area had no idea that they had diabetes*,* and I feel good that they have started taking their medicines now because I found out first that they have diabetes.” (ASHA*,* Interview*,* Female*,* Rohtak*,* Haryana)*.

Physicians also expressed satisfaction with the training, particularly the app-based format, which accommodated their busy schedules. An experienced physician acknowledged the value of the system for younger doctors:

*“I also like the functionality of the CDSS where I was being advised what medications to give… while I have more than 20 years of experience*,* for a young PHC doctor*,* this would be quite helpful.” (Physician*,* Interview*,* Male*,* Guntur*,* Andhra Pradesh)*.

### Usefulness of the intervention

The participants appreciated the usefulness and benefits of both the CDSS and the overall intervention strategy. Physicians emphasized the potential for scalability and impact of this CDSS and recommended wider adoption.

*“The mobile-based CDSS was really effective*,* and I encourage this to be scaled up to other PHCs… it is important that… other doctors are also able to use it.” (Physician*,* Interview*,* Male*,* Guntur*,* Andhra Pradesh)*.

The comprehensive nature of the intervention, which included lifestyle advice and patient education resources, was also commended.

*“Even the lifestyle advice is quite useful. I saw that the ASHA module contains videos and other personal risk factor-specific information and some resources on quitting some risky behaviours… I found it effective and useful for them.” (Physician*,* Interview*,* Male*,* Rohtak*,* Haryana)*.

ASHAs also emphasized the usefulness of the CDSS in enhancing their ability to collect, track, and utilize patient data.

*“This tool was very useful. We would not have been able to do this work on paper or collect this information effectively. Now this information is available with us*,* and we can use it for the benefit of these patients.” (ASHA*,* FGD*,* Female*,* Guntur*,* Andhra Pradesh)*.

The ability to access and compare previous visit information facilitated more informed patient interactions and personalized advice. Furthermore, one of the ASHAs mentioned.

*“The interactive ‘what if’ module*,* which visually demonstrated the potential health benefits of lifestyle changes*,* proved to be a powerful tool for patient motivation.” (ASHA*,* Interview*,* Female*,* Rohtak*,* Haryana)*.

## Feasibility

### Barriers to implementation

The ASHAs were apprehensive of the increased workload on top of their existing responsibilities in maternal and child health, immunization, and other community health programs.

*“Our workload is very high… sometimes it becomes too much*,* and on top of it*,* we also have household responsibilities to take care of… sometimes it gets busy*,* and we just stop being able to manage our household chores well.” (ASHA*,* Interview*,* Female*,* Guntur*,* Andhra Pradesh)*.

ASHAs expressed concerns about the continuity of the program after the study period ended because of the absence of a long-term strategy.

*“While we are in the study*,* we are also getting some support from our medical officer and auxiliary nurse midwife (ANM)*,* but the moment the study ends*,* we will have to return all the equipment back*,* and we will stop doing this work for our village*,* which is a problem.” (ASHA*,* FGD*,* Female*,* Rohtak*,* Haryana)*.

Limited access to long-term medication supplies was identified as a challenge. According to one participant:

*“Despite being affiliated with this project and being screened*,* I am not getting the medicines for a longer duration of time… the government needs to make sure that I get them… they should give diabetes medicine for a longer period of time*,* at least 3 to 4 months.” (Participant*,* FGD*,* Female*,* 55*,* Rohtak*,* Haryana)*.

### Facilitators to implementation

Strong support from district-level medical officers and supervisors was identified as a key enabler. An ASHA described the importance of this support:

*“Because this program… had the support of the district-level medical officer and my seniors… I got the support*,* and they were also key for me to learn the skills*,* and without interruptions*,* I could work.” (ASHA*,* Interview*,* Female*,* Guntur*,* Andhra Pradesh)*.

### Impact of the intervention on staff knowledge and skills

ASHAs reported increased confidence and competence in diabetes management. Community members recognized the improved capacity of ASHAs to provide essential health services.

*“We saw ASHAs taking care of doing the blood glucose screening and BP screen*,* and we found it very useful that there is someone close by who knows about all these things*,* and we do not have to go far for these basic checkups.” (Participant*,* Interview*,* Male*,* 61*,* Guntur*,* Andhra Pradesh)*.

Physicians also observed a significant improvement in the knowledge and skills of ASHAs.

*“I was amazed to see the kind of data that has been collected by the ASHAs… I did not believe that… one of them… had collected the status*,* and she was like*,* yes*,* she also did show me the pictures of the various events that she had gone to and demonstrated the blood glucose screening.” (Physician*,* Interview*,* Male*,* Rohtak*,* Haryana)*.

### Impact of the intervention on usual work routines

Physicians acknowledged the usefulness of the CDSS in providing patient history and informing treatment decisions but also noted challenges in integrating its use into their busy outpatient department (OPD) routines.

*“While this tool is helpful*,* it is important to understand that we have a running OPD. In busy times*,* it is difficult to enter data on the mobile tablet and guide the patient in real-time… if I have a few people waiting for me outside*,* then using it is a bit of a challenge…” (Physician*,* Interview*,* Female*,* Guntur*,* Andhra Pradesh)*.

Some ASHAs highlighted the need for flexibility in scheduling intervention activities.

*“I have never faced any challenge myself because the work done is quick… but it is just that others found this difficult because they had more immunization duty responsibilities*,* and on some days*,* especially on Wednesdays*,* it became difficult. That is why we did not do any IMPACT Diabetes-related work on Wednesdays.” (ASHA*,* FGD*,* Female*,* Rohtak*,* Haryana)*.

Involvement of and support from their direct supervisors (ANMs), played a significant role in the smooth integration of the intervention into their work routines.

*“While some of the ANMs were supportive of this task*,* the ANMs were not fully involved in or fully informed about the study initially*,* and that is why we had a problem with scheduling the task… it was suggested that… ANMs should also be given some initial idea… so that the performing of the day-to-day activities is not difficult for the ASHAs.” (ASHA*,* FGD*,* Female*,* Guntur*,* Andhra Pradesh)*.

### Resource requirements and allocation

ASHAs participating in FGDs reported that they received all the necessary resources, including mobile tablets, blood glucose meters, BP machines, and adequate supplies, to effectively perform their duties. One ASHA commented:

*“There were no problems with the resources that were given to us… the supplies were never short… the equipment was available*,* and the BP machine also worked fine.” (ASHA*,* FGD*,* Female*,* Guntur*,* Andhra Pradesh)*.

## Discussion

This study demonstrates the feasibility of implementing a multi-component, technology-based intervention to improve diabetes screening and management within the primary healthcare system in India. The intervention was successfully integrated into the workflows of ASHAs and physicians, despite their varying backgrounds and prior experience with technology leading to a clinically meaningful reduction in HbA1c (≥ 0·5%), increased physician visits and facilitated greater use of appropriate anti-diabetic medications compared to the control group. The successful training and engagement of ASHAs in utilizing the CDSS for data collection, patient education, and communication with physicians highlights the potential for leveraging technology to empower community health workers in diabetes care. This is consistent with studies that have shown the successful training and engagement of community health workers in utilizing technology for various healthcare needs, including non-communicable diseases, maternal and child health, and infectious diseases^27–29^.

The preliminary effectiveness estimates from this study showed a significant difference in glycaemic control between the intervention and control groups, with a higher proportion of patients in the intervention group achieving a clinically meaningful reduction in HbA1c (≥ 0·5%) compared to the control group. This finding aligns with studies that have demonstrated the positive impact of technology-enabled interventions on diabetes management. Esferjani et al. (2020) found that a mobile-based educational intervention improved self-care practices and reduced HbA1c levels among elderly individuals with type II diabetes in Iran^30^. Gerber et al. (2023) reported significant improvements in HbA1c levels among African American patients with type II diabetes who received mHealth intervention delivered by clinical pharmacists and health coaches^31^. These studies, along with others, suggest that mHealth interventions can be effective in improving management of individuals with diabetes^32^. In contrast, Prabhakaran et al. found no significant difference between the intervention and enhanced usual care in terms of BP or glycaemic control in a cluster randomised trial of mHealth intervention in rural India^33^. Factors such as intervention design, target population, implementation context, and the specific outcomes measured can influence the effectiveness of these multicomponent interventions^34^. Our intervention’s success may be attributed to its comprehensive design, which included hands-on training for ASHAs and physicians, the provision of a robust CDSS for real-time decision-making, and integration within existing primary healthcare workflows.

While a higher proportion of intervention participants achieved a clinically meaningful HbA1c reduction, average HbA1c levels remained similar between groups (8.6% vs. 8.8%) and relatively high. This may reflect the short intervention duration (9 months), severity of baseline diabetes, or the need for intensive therapy beyond ASHA-led support.

Qualitative findings revealed high levels of patient satisfaction with the intervention, particularly realizing the comprehensive and accessible care, the integration of technology, and the continuity of care facilitated by the CDSS. ASHAs and physicians reported positive experiences with the intervention, expressing a sense of empowerment, enhanced professional development, and the usefulness of the CDSS in facilitating informed decision-making and patient management. The findings from this study add to the existing evidence by demonstrating the acceptability and feasibility of integrating these approaches within the existing primary healthcare system in India^22,28^. Feedback from ASHAs and PHC physicians highlighted the importance of ongoing efforts to manage workload and optimize workflows. The addition of new responsibilities, such as diabetes care, to ASHAs’ already extensive duties raises important concerns about potential overburdening and the unintended dilution of their traditional roles. In this study, several strategies were implemented to address this risk, including comprehensive training, the use of streamlined digital tools such as a CDSS for efficient data collection, and flexible scheduling of intervention activities, as reported by the ASHAs.

The SMARThealth CDSS used in this study is specifically designed to support the integrated management of multiple NCDs, including hypertension, as well as maternal and child health responsibilities. The system was developed based on behaviour change theory to support frontline health workers in delivering evidence-based care and facilitating patient engagement^22,28^. This platform enables a more holistic approach to care delivery and offers structured decision support, which is often lacking in general platforms. In addition to data collection and screening, this multi-component intervention also facilitates referral tracking to PHC doctors and supports ASHAs in delivering audio-visual interventions on behavioural risk factors directly to community members. Implementing such a comprehensive strategy, along with efforts to increase the number of frontline health workers helps prevent overburdening ASHAs and to maximize their impact across the NCD spectrum.

Several features of the IMPACT Diabetes intervention contributed to its success. While the overall intervention was pragmatically designed and heavily contextualized during the formative phase through consultation with state health department officials, academics, ASHAs, ANMs, and PHC physicians, ensuring its integration into existing workflows. The intervention was delivered by existing ASHAs and physicians without introducing additional personnel, enhancing its acceptability within the existing system. This participatory approach, combined with comprehensive training, empowered ASHAs to effectively utilize technology for data collection, patient education, and communication with physicians, ultimately contributing to improved diabetes management.Another strength lies in the study’s focus on essential data collection elements, limiting assessments to those directly relevant to the CVD risk algorithm. This streamlined approach improved efficiency and ensured a high rate of data completion, minimizing missing data and potential bias. To minimize contamination, the study used a cluster randomized design, randomizing villages or peri-urban areas within each PHC.

Despite these promising findings, several challenges can hinder the scalability and long-term sustainability of the IMPACT Diabetes intervention. The ASHA-led screening of approximately 100 community members, while demonstrating effective implementation at the micro level, highlights that scaling the program to cover the larger population with the current workforce would have significant resource implications. Currently, no long-term strategy exists to ensure continued support for ASHAs and maintain their engagement beyond the study period. The lower number of visits observed in the usual care arm underscores the potential influence of the ASHA reminders on adherence and highlights the need for further evaluation in a larger-scale trial to assess long-term adherence patterns. Additionally, consistent availability of essential medications, a key feature highlighted by participants and a likely contributor to the observed reduction in HbA1c values, remains a concern in routine practice after the intervention concludes. The study was conducted in only two districts, which may not adequately represent the vast diversity of health systems and cultural practices across India, thereby limiting the generalizability of the findings. Future research should consider the influence of social factors, income, literacy, and digital resources, which are crucial for evaluating the equitable reach and impact of mHealth-led approaches.

The strengths of the study include involvement of multiple stakeholders, including ASHAs and primary healthcare physicians, which ensured that the intervention was integrated into the existing healthcare system and aligned with routine care practices. This pragmatic approach enhanced the feasibility and acceptability of the intervention, as highlighted by the high participation rates and positive feedback from healthcare providers. Furthermore, as a multi-component intervention, it is challenging to isolate the specific contribution of each component to the observed outcomes. While subgroup analyses (e.g., by gender, age, baseline HbA1c, socioeconomic status, or district) would provide valuable insights into differential effects, they were beyond the scope of this preliminary effectiveness study.

Future studies should aim to separate the individual effects of the CDSS, training, ASHA support, and access to lab testing to optimize intervention design and resource allocation and explore other clinical indicators such as blood pressure and lipid profiles at endpoint to provide further insights into the intervention’s broader impact.

## Conclusions

The IMPACT Diabetes study demonstrated the feasibility and effectiveness of a community-based, technology-enabled intervention that combines task-shifting with a mobile CDSS to improve diabetes identification and management in India. It led to significantly higher proportion of participants achieving a clinically meaningful reduction (≥ 0.5%) in HbA1c, alongside improvements in healthcare utilization, and medication use, while also being well-received by patients, ASHAs, and physicians. For long-term sustainability and maximal public health impact, such interventions require formal integration into national programs, ensuring continuous training, supervision for ASHAs, and reliable medicine availability. Future research should evaluate individual components, assess cost-effectiveness, and explore adaptability to resource-constrained settings.

## Data Availability

The datasets generated and/or analysed during the current study are available from the corresponding author on reasonable request.

## Abbreviations

ASHA: Accredited Social Health Activist
BP: Blood Pressure
CDSS: Clinical Decision Support System
CVD: Cardiovascular Disease
FGD: Focus Group Discussion
HbA1c: Glycated haemoglobin
HWC: Health and Wellness Center
IMPACT Diabetes: Innovative mobile-health-led participatory approach to comprehensive screening and treatment of diabetes
mHealth: mobile Health
OPD: Outpatient Department
PHC: Primary Health Center
SMARThealth: Systematic Medical Appraisal, Referral and Treatment
